# The Endosymbiotic Bacterium *Wolbachia* Selectively Kills Male Hosts by Targeting the Masculinizing Gene

**DOI:** 10.1371/journal.ppat.1005048

**Published:** 2015-07-14

**Authors:** Takahiro Fukui, Munetaka Kawamoto, Keisuke Shoji, Takashi Kiuchi, Sumio Sugano, Toru Shimada, Yutaka Suzuki, Susumu Katsuma

**Affiliations:** 1 Department of Agricultural and Environmental Biology, Graduate School of Agricultural and Life Sciences, The University of Tokyo, Bunkyo-ku, Tokyo, Japan; 2 Department of Medical Genome Sciences, Graduate School of Frontier Sciences, The University of Tokyo, Minato-ku, Tokyo, Japan; 3 Department of Computational Biology, Graduate School of Frontier Sciences, The University of Tokyo, Kashiwa, Chiba, Japan; Monash University, AUSTRALIA

## Abstract

Pathogens are known to manipulate the reproduction and development of their hosts for their own benefit. *Wolbachia* is an endosymbiotic bacterium that infects a wide range of insect species. *Wolbachia* is known as an example of a parasite that manipulates the sex of its host's progeny. Infection of *Ostrinia* moths by *Wolbachia* causes the production of all-female progeny, however, the mechanism of how *Wolbachia* accomplishes this male-specific killing is unknown. Here we show for the first time that *Wolbachia* targets the host masculinizing gene of *Ostrinia* to accomplish male-killing. We found that *Wolbachia*-infected *O*. *furnacalis* embryos do not express the male-specific splice variant of *doublesex*, a gene which acts at the downstream end of the sex differentiation cascade, throughout embryonic development. Transcriptome analysis revealed that Wolbachia infection markedly reduces the mRNA level of *Masc*, a gene that encodes a protein required for both masculinization and dosage compensation in the silkworm *Bombyx mori*. Detailed bioinformatic analysis also elucidated that dosage compensation of Z-linked genes fails in *Wolbachia*-infected *O*. *furnacalis* embryos, a phenomenon that is extremely similar to that observed in *Masc* mRNA-depleted male embryos of *B*. *mori*. Finally, injection of *in vitro* transcribed *Masc* cRNA into *Wolbachia*-infected embryos rescued male progeny. Our results show that *Wolbachia*-induced male-killing is caused by a failure of dosage compensation via repression of the host masculinizing gene. Our study also shows a novel strategy by which a pathogen hijacks the host sex determination cascade.

## Author Summary

Pathogens are known to manipulate the physiology, behavior, and reproduction of their hosts for their own benefit. The endosymbiotic bacterium *Wolbachia* is known to manipulate the sex of its host's progeny. Male-killing is one of the phenotypes that *Wolbachia* induces, but the mechanism of how *Wolbachia* induces sex-specific death is unknown. Here we found a marked down-regulation of *Masc*, a lepidopteran-specific zinc finger protein gene, in embryos that are produced by *Wolbachia*-infected *Ostrinia* moths. We also observed that dosage compensation fails in *Wolbachia*-infected *Ostrinia* embryos. The findings of this study and our previous study using a lepidopteran model insect *Bombyx mori* indicate that *Wolbachia* has evolved to hijack the *Masc*-dependent, lepidopteran insect-specific sex determination system by capturing an unknown factor during *Wolbachia*-host coevolution.

## Introduction

*Wolbachia* is a genus in Rickettsiales, a diverse order of intracellular bacteria. Recent meta-sequencing analysis shows that over 65% of insect species possess *Wolbachia* [1], indicating that *Wolbachia* is the most widespread and common intracellular bacterium that infects insects. *Wolbachia* is a well-known example of a parasite that alters host reproduction to facilitate its own propagation. *Wolbachia*-induced phenotypes include parthenogenesis, feminization, cytoplasmic incompatibility, and male-killing, each of which is supposed to be adaptive for *Wolbachia* by enhancing the production of infected females [2].

*Wolbachia*-induced male-killing has been reported in three insect orders, Coleoptera [3], Diptera [4], and Lepidoptera [5], in which male-killing occurs mainly during embryogenesis. Recent advances in *Wolbachia*-induced male-killing have been made mainly from studies using *Ostrinia* moths [5–7]. *Ostrinia* species (corn borer) have a WZ/ZZ sex chromosome system and all of the progeny of *Wolbachia*-infected mother moths have a W chromosome, indicating the existence of male-killing [7]. The male-type splice variant of *doublesex* (*dsx*), a gene that acts at the downstream end of the sex differentiation cascade [8–9], is not detected in *Ostrinia* late embryos that originate from *Wolbachia*-infected mothers [7], suggesting that *Wolbachia*’s sexual manipulation is presumably established at an early embryonic stage. However, the molecular mechanism(s) by which *Wolbachia* manipulates sex and male lethality in *Ostrinia* moths has remained elusive.

The sex determination cascade in lepidopteran insects has been studied mainly using silkworm *Bombyx mori* as a model insect [10–11]. In *B*. *mori*, females have ZW sex chromosomes and males have two Z chromosomes. *B*. *mori* femaleness is strongly determined by the presence of the W chromosome irrespective of the Z chromosome number, suggesting that there is a dominant feminizing gene (*Fem*) on the W chromosome [12–13]. In 2014, we discovered that *Fem* is a precursor of a single W chromosome-derived PIWI-interacting RNA (piRNA) [14]. We also identified the target gene of *Fem*-derived piRNA (*Fem* piRNA), which is located on the Z chromosome. Depletion of this Z-linked gene, *Masculinizer* (*Masc*), in male embryos leads to the production of the female-type splicing of *B*. *mori dsx*, indicating that the product of *Masc* is a masculinizing factor. These results revealed that sex of *B*. *mori* is determined by the *Fem* piRNA-*Masc* cascade [14].

Further experiments showed that silencing of *Masc* in *B*. *mori* embryos results in male-specific lethality [14]. Deep sequencing (RNA-seq) of *Masc* mRNA-depleted embryos revealed that the Masc protein is required for the repression of global transcription from the Z chromosome in male embryos, and that a failure of this dosage compensation causes male-specific embryonic death [14]. Bioinformatic analysis also revealed that most of the Z-linked genes are dosage compensated by 72 hours post-oviposition (hpo) [15]. These results indicate that *Masc*-mediated control of dosage compensation at an early embryonic stage is essential for male development of *B*. *mori*.

In this study, we attempted to explore the mechanism by which *Wolbachia* accomplishes sexual manipulation using *Wolbachia*-infected *Ostrinia* moths as a model. We previously showed that artificial depletion of *Masc* mRNA in *B*. *mori* early embryos results in male-specific embryonic lethality [14]. This phenomenon likely mimics the way that *Wolbachia* induces male-killing in *Ostrinia* [7]. In the light of these facts, we first focused on the expression of *Ostrinia* homologue of *Masc* in *Wolbachia*-infected embryos. Transcriptome analysis by RNA-seq revealed that *Wolbachia*-induced male-killing is established by a failure of dosage compensation through *Masc* mRNA depletion. Furthermore, injection of *Masc* cRNA into *Wolbachia*-infected *Ostrinia* embryos prevented males from being killed at embryonic stages. This is the first study to identify the *Wolbachia*’s target that is utilized for sexual manipulation.

## Results and Discussion

### Characterization of a male-killing *Wolbachia* found in *Ostrinia* moths

**Fig 1:**
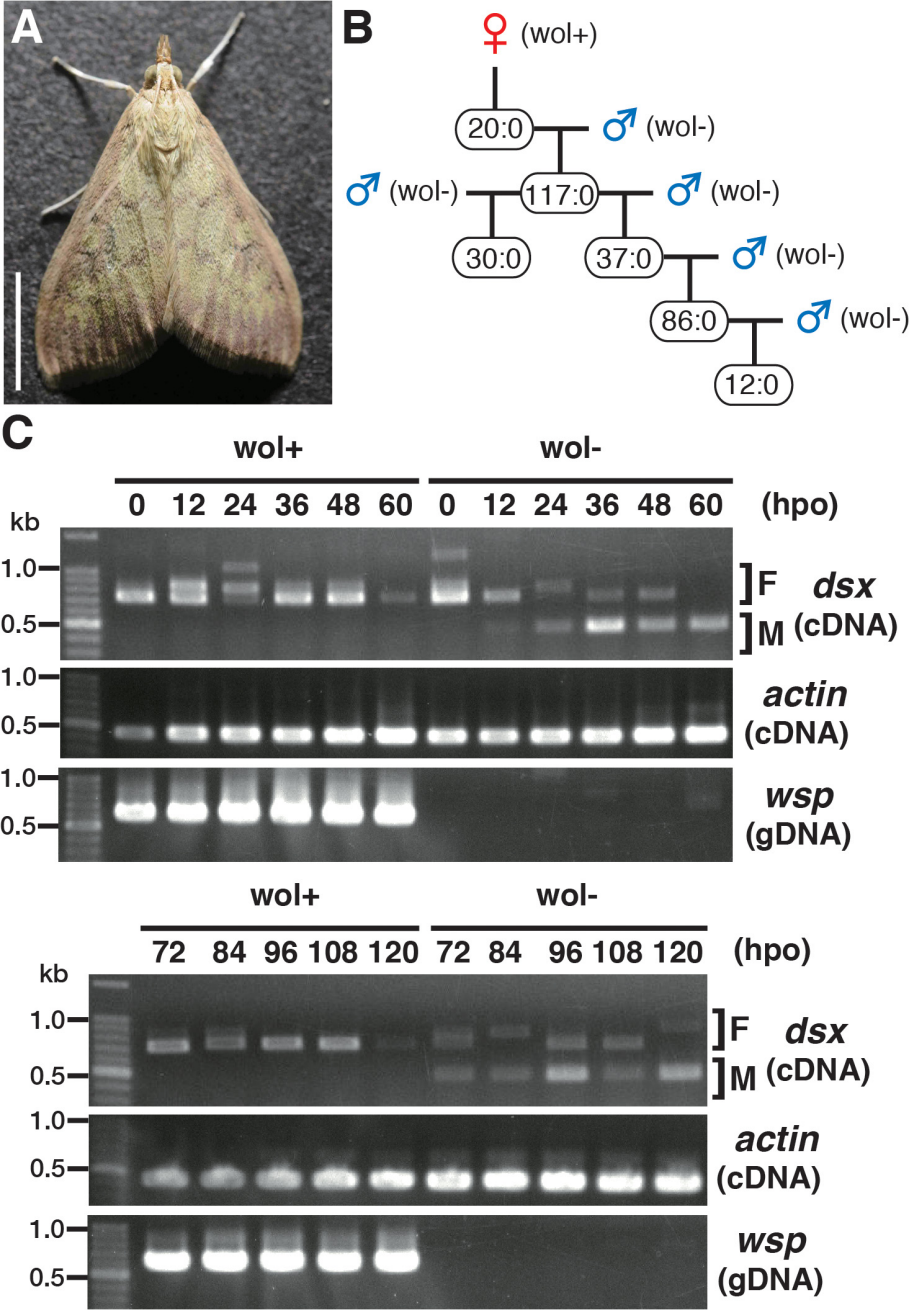
Characterization of a male-killing *Wolbachia* found in adult *Ostrinia*. (A) *Wolbachia*-infected female moth of *Ostrinia*. Bar, 5 mm. (B) Brood sex ratios in a *Wolbachia*-infected matriline through 5 generations. The female:male ratio of each mating is shown. (C) Splicing patterns of *Ostrinia dsx* during embryonic development (0–120 hpo) of embryos that were infected with *Wolbachia* (wol+) or uninfected (wol-). Total RNA was prepared from *Ostrinia* embryos (25–50 embryos at each time point) and subjected to RT-PCR for *dsx* and *actin*. The F and M indicate female- and male-type splicing of *Ostrinia dsx*, respectively. *actin* was used as an internal control. *Wolbachia* infection was verified by PCR using *wsp*-specific primers. Note that in other experiments (S1E Fig), the female-type splicing variants were clearly detected when the same RNA pool prepared from *Wolbachia*-uninfected embryos at 60 hpo was used, although that was not observed in this case.

We collected over 80 *Ostrinia* moths in the field and found 3 female moths that were infected with *Wolbachia* (Fig 1A). One of these moths produced only female progeny. By sex pheromone analysis, this female was found to be *O*. *furnacalis*. Subsequently, when the females were mated with other *Wolbachia*-free *O*. *furnacalis* males only female progeny were produced. There was no deviation in this pattern of female only progeny through 5 generations (in total 302 females and no males judged by external morphology at the adult stage) (Fig 1B).

We established the quantitative PCR (qPCR)-based molecular sexing method for *O*. *furnacalis* (S1A Fig), and verified that *Wolbachia*-infected moths were all female (S1B Fig). Using this method, we found that *Wolbachia*-infected embryos (just prior to hatching) contained both female and male individuals (S1C Fig). However, the hatched larvae were all female (S1C Fig), indicating that this *Wolbachia* induces male-specific embryonic lethality. In addition, when *Wolbachia* was eliminated from infected individuals by tetracycline treatment and *Wolbachia*-eliminated female moths were mated with other *Wolbachia*-free males, only male progeny were produced (S1D Fig). Taken together with these results, we concluded that this *Wolbachia* strain induces male-killing in *O*. *furnacalis*, which is similar to the phenotype observed in *Wolbachia*-infected *O*. *scapulalis* [7].

Sugimoto and Ishikawa [7] reported that the male-type splice variants of *Ostrinia dsx* [6] is not expressed in all 5-day-old *O*. *scapulalis* embryos (just prior to hatching) that are infected with a male-killing *Wolbachia*. In order to determine the precise developmental stage at which *Wolbachia* starts sexual manipulation in *Ostrinia*, we examined the splicing patterns of *dsx* in *Wolbachia*-infected *Ostrinia* embryos starting immediately after oviposition. Both male- and female-type variants of *dsx* were observed in uninfected embryos (Fig 1C). The male-type variant in uninfected embryos was detected from 12 hpo, indicating that the sex determination signal is transmitted prior to 12 hpo in *Ostrinia*. In contrast, the male-type *dsx* variant was not detected in *Wolbachia*-infected embryos throughout embryogenesis (Fig 1C). These results indicate that *Wolbachia* manipulates the sex of *Ostrinia* from the beginning of the sex determination period.

### Marked down-regulation of *Masc* in *Wolbachia*-infected *Ostrinia* embryos

**Fig 2:**
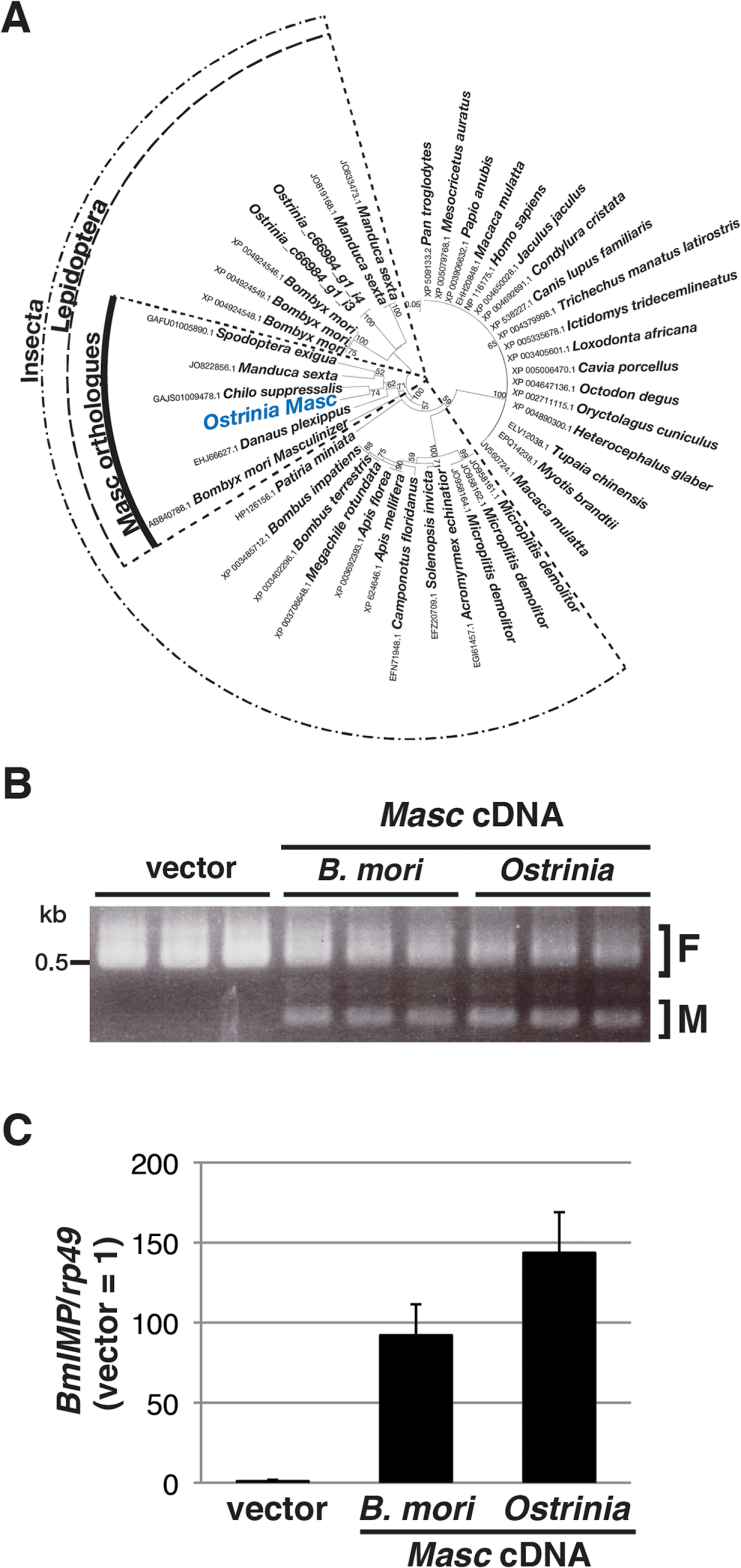
Identification and characterization of *Ostrinia* Masc protein. (A) Phylogenetic analysis of *Ostrinia* Masc protein. The neighbor-joining tree was generated using the amino acid sequences corresponding to the zinc finger domains from 43 proteins showing homology to *B*. *mori* Masc protein. The numbers on the internal branches represent the support value following bootstrap analysis (1,000 replicates). (B) Effect of *Ostrinia Masc* cDNA transfection on the *Bmdsx* splicing in BmN4 cells. The splicing patterns of *Bmdsx* in BmN4 cells transfected with *Ostrinia* or *B*. *mori Masc* expression vectors or empty vector were examined by RT–PCR. The F and M indicate female- and male-type splicing of *Bmdsx*, respectively. (C) Effect of *Ostrinia Masc* cDNA transfection on *BmIMP* expression. *BmIMP* expression was examined by RT-qPCR. *rp49* was used as a normalization control. Data shown are means + standard deviation of triplicates.

In order to investigate what occurs during *Wolbachia*-induced sexual manipulation, we performed RNA-seq experiments using RNAs prepared from *Wolbachia*-infected and-uninfected embryos (of both sexes) at 0, 12, 24, 36, and 48 hpo. We recently showed that Masc protein encodes a lepidopteran-specific CCCH-tandem zinc finger protein, which is required for both masculinization and dosage compensation in *B*. *mori* [14]. RNA interference-mediated knockdown experiments also show that depletion of *Masc* mRNA in *B*. *mori* early embryos results in male-specific death via a failure of dosage compensation [14]. In order to test whether this male-specific death in *B*. *mori* is related to the *Wolbachia*-induced male killing in *Ostrinia*, we first identified and characterized the *Masc* homolog of *Ostrinia*. Using RNA-seq data, we identified contigs that potentially encode a protein with significant homology to Masc proteins (Fig 2A and S2 Fig). *Ostrinia* Masc was composed of 583 amino acid residues and showed 28.1% identity to *B*. *mori* Masc (S2 Fig). Phylogenetic analysis based on the amino acid sequences of zinc finger domains revealed that *Ostrinia* Masc was closely related to that of *Chilo suppressalis* (Fig 2A).

The silkworm ovarian cell line BmN4 expresses the female-type *Bmdsx* variant only, whereas transfection of *B*. *mori Masc* cDNA results in the production of the male-type splice variant [14]. Using this system, we examined whether *Ostrinia* Masc protein also has a masculinizing activity. As shown in Fig 2B, we observed the expression of the male-type variant of *Bmdsx* in BmN4 cells when transfected with *Ostrinia Masc* cDNA as well as *B*. *mori Masc* cDNA. In addition, we examined the expression of *B*. *mori insulin-like growth factor II mRNA-binding protein* (*BmIMP*), a factor that is involved in the male-specific splicing of *Bmdsx* [16], in *Ostrinia Masc* cDNA-transfected cells. We found that either *B*. *mori* or *Ostrinia Masc* cDNA markedly enhanced *BmIMP* expression (Fig 2C). These results strongly suggest that *Ostrinia Masc* encodes a masculinizing protein and may be required for masculinization in *Ostrinia* sex determination pathway.

**Fig 3:**
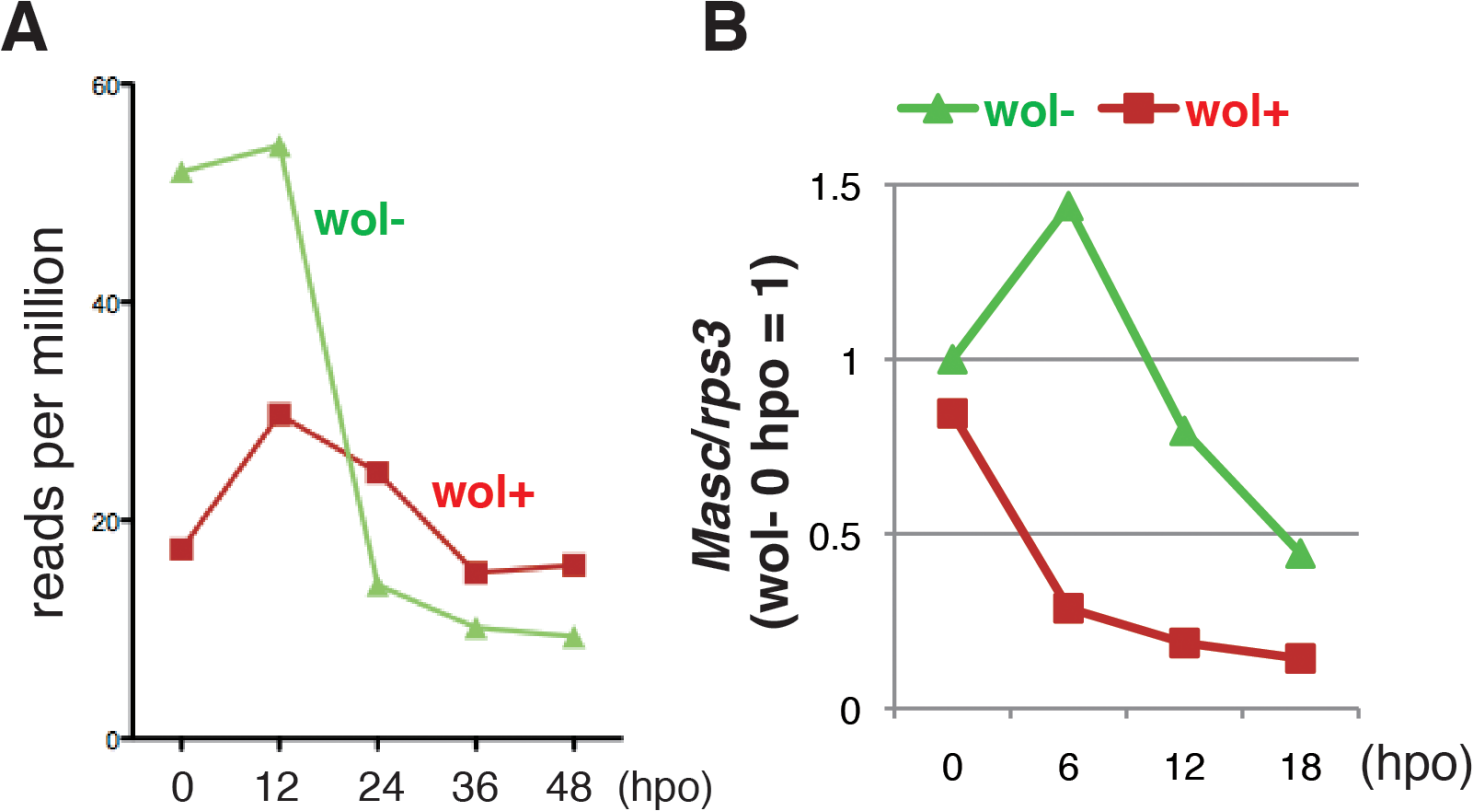
A marked decrease in *Masc* mRNA was observed in *Wolbachia*-infected embryonic *Ostrinia*. (A) Number of *Ostrinia Masc* coding sequence-derived tags found in RNA-seq library generated from *Wolbachia*-infected (wol+) and-uninfected (wol-) embryos at 0, 12, 24, 36, and 48 hpo. (B) Expression profile of *Ostrinia Masc* mRNA during early embryogenesis (0, 6, 12, and 18 hpo). Total RNA was prepared from embryos (25–50 embryos at each time point) that were wol+ or wol-. *Rps3* was used as a normalization control for RT-qPCR.

We next compared the level of *Masc* mRNA in *Wolbachia*-infected and-uninfected embryos by mapping RNA-seq tags onto the *Ostrinia Masc* coding sequence. We found a significant decrease in *Masc* mRNA in *Wolbachia*-infected embryos prior to 12 hpo (Fig 3A). To elucidate this reduction in greater detail, we performed reverse transcription-qPCR (RT-qPCR) using total RNA isolated from *Wolbachia*-infected and uninfected embryos at 0, 6, 12, and 18 hpo. In uninfected embryos, *Masc* expression peaked at 6 hpo and decreased rapidly. In contrast, *Masc* expression in *Wolbachia*-infected embryos declined by 6 hpo, and remained at a low level compared with that observed in uninfected embryos (Fig 3B). These results clearly showed that *Wolbachia* infection markedly reduces *Masc* mRNA level during embryogenesis of *Ostrinia*. Together with transfection results (Fig 2B and 2C) and our previous results showing that knock down of *Masc* mRNA results in the production of the female-type *dsx* in male embryos of *B*. *mori* [14], we conclude that the lack of the male-type *dsx* in *Wolbachia*-infected *Ostrinia* embryos (Fig 1C) was caused by down-regulation of *Masc* mRNA (Fig 3A and 3B).

### Failure of dosage compensation in *Wolbachia*-infected *Ostrinia* embryos

**Fig 4:**
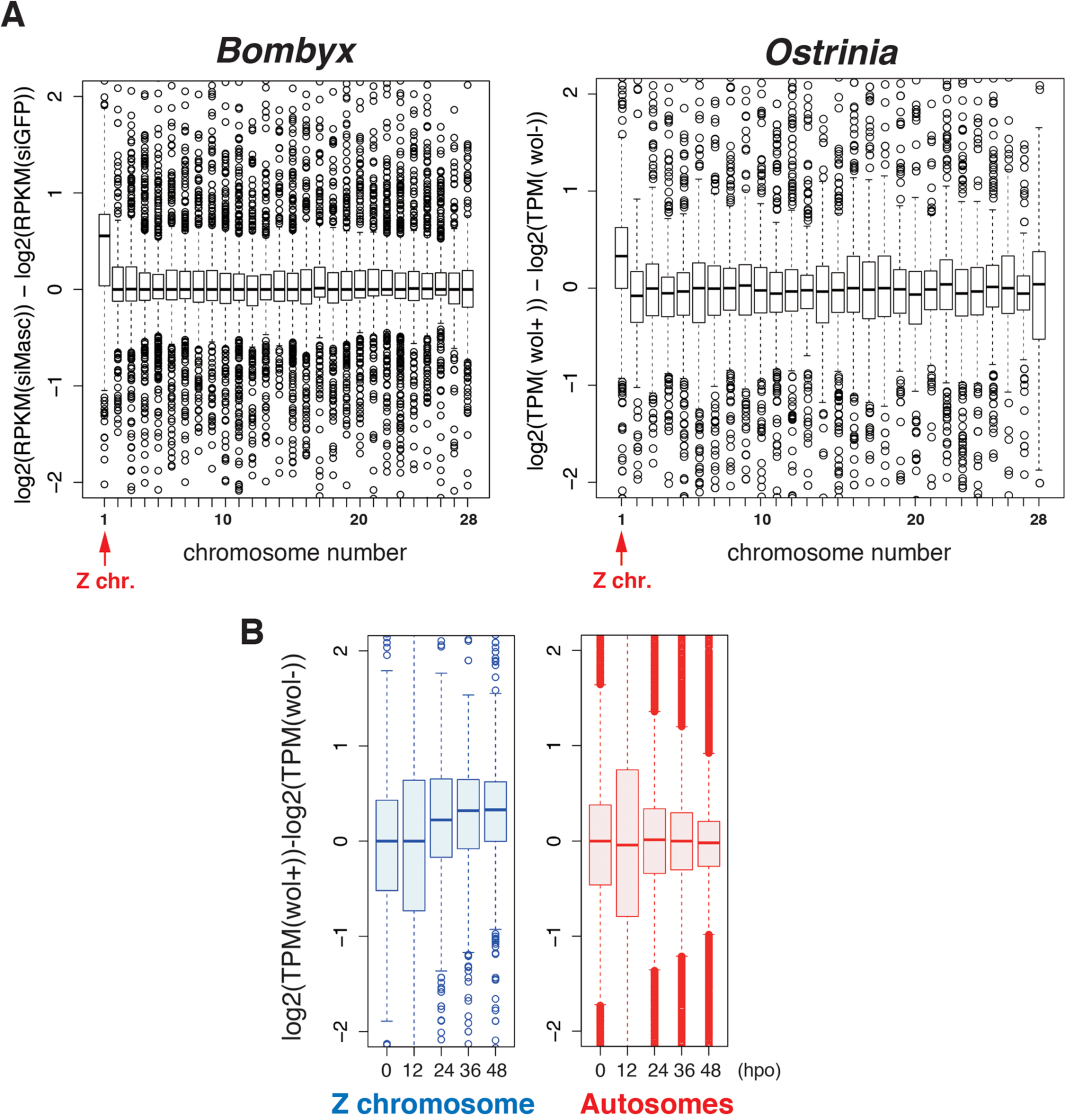
A failure of dosage compensation in *Wolbachia*-infected embryos. (A) Chromosomal distribution of differentially expressed transcripts in *B*. *mori* and *Ostrinia* embryos. The *B*. *mori* RNA-seq data of *Masc* RNAi experiments (*GFP* and *Masc* RNAi embryos of mixed sex, 72 h post-injection) were used as a control experiment (left panel). The *Ostrinia* RNA-seq data (*Wolbachia*-infected and uninfected embryos of both sexes at 48 hpo were used to draw the panel on the right. The chromosome number for each *Ostrinia* transcript-derived contig was assigned using *B*. *mori* gene models. The data are shown by *box-and-whisker* diagrams. The boxes represent the median and 25–75 percentile ranges of the expression ratios. (B) Time-course and Z chromosome-biased distribution of differentially expressed transcripts in *Wolbachia*-infected and uninfected *Ostrinia* embryos. The data are separately shown in Z chromosome (blue)- and autosome (red)-linked genes. The data are shown by *box-and-whisker* diagrams. The boxes represent the median and 25–75 percentile ranges of the expression ratios.

Considering our recent finding that depletion of *Masc* mRNA in early embryos of *B*. *mori* results in male-specific embryonic lethality due to a failure of dosage compensation [14], we hypothesized that a decrease in *Masc* mRNA in *Wolbachia*-infected *Ostrinia* embryos also affects dosage compensation, presumably resulting in a male-specific embryonic death. To test this hypothesis, we examined dosage compensation effects in *Wolbachia*-infected *Ostrinia* embryos using RNA-seq data. As reported previously [14], Z-linked transcripts are expressed at higher levels in *Masc* mRNA depleted *B*. *mori* embryos than in control embryos (Fig 4A, left panel). A similar transcriptional bias in putative Z-linked genes in *Wolbachia*-infected *Ostrinia* embryos at 48 hpo was also found (Fig 4A, right panel). A failure of dosage compensation of Z-linked genes was detected from 24 hpo and continued to 48 hpo (Fig 4B and S3 Fig). These results strongly support our hypothesis that *Wolbachia* infection leads to abnormally enhanced expression of Z-linked genes in male embryos via *Masc* mRNA down-regulation, resulting in a male-killing phenotype.

### Rescue of male progeny by injection of *Masc* cRNA into *Wolbachia*-infected *Ostrinia* embryos

**Fig 5:**
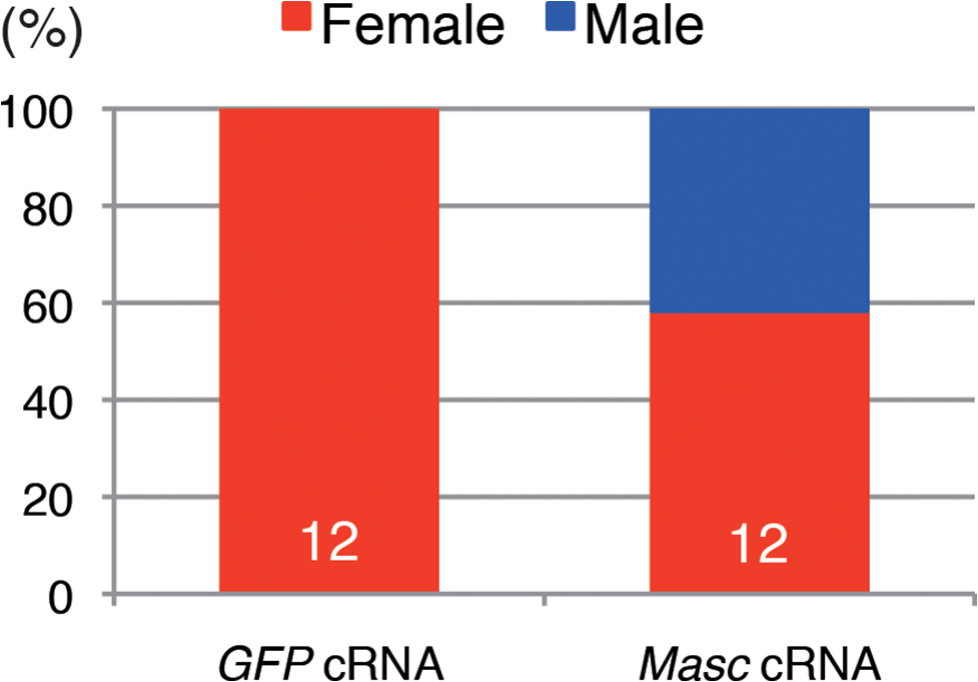
Injection of *Masc* cRNA rescued the male-specific death of *Wolbachia*-infected embryonic *Ostrinia*. Capped, poly(A)-tailed *Masc* cRNA was synthesized and injected into *Wolbachia*-infected embryos immediately after oviposition. The hatched larvae were collected and molecularly sexed. The number indicates the sample size of each group.

To obtain direct evidence that down-regulation of *Masc* mRNA in *Wolbachia*-infected *Ostrinia* embryos results in the male-killing phenotype, we performed rescue experiments by injecting *in vitro* synthesized capped, poly(A)-tailed *Masc* cRNA into *Wolbachia*-infected embryos. As shown in Fig 5, the hatched larvae injected with control (*GFP*) cRNA were all female, whereas both male and female larvae were observed when injected with *Masc* cRNA. This clearly indicates that introduction of *Masc* cRNA into *Wolbachia*-infected embryos can rescue male embryos. Together with the results of transcriptome data, we conclude that a decrease in *Masc* mRNA in *Wolbachia*-infected embryos causes a male-killing phenotype via a failure of dosage compensation.

### Conclusion and perspective

**Fig 6:**
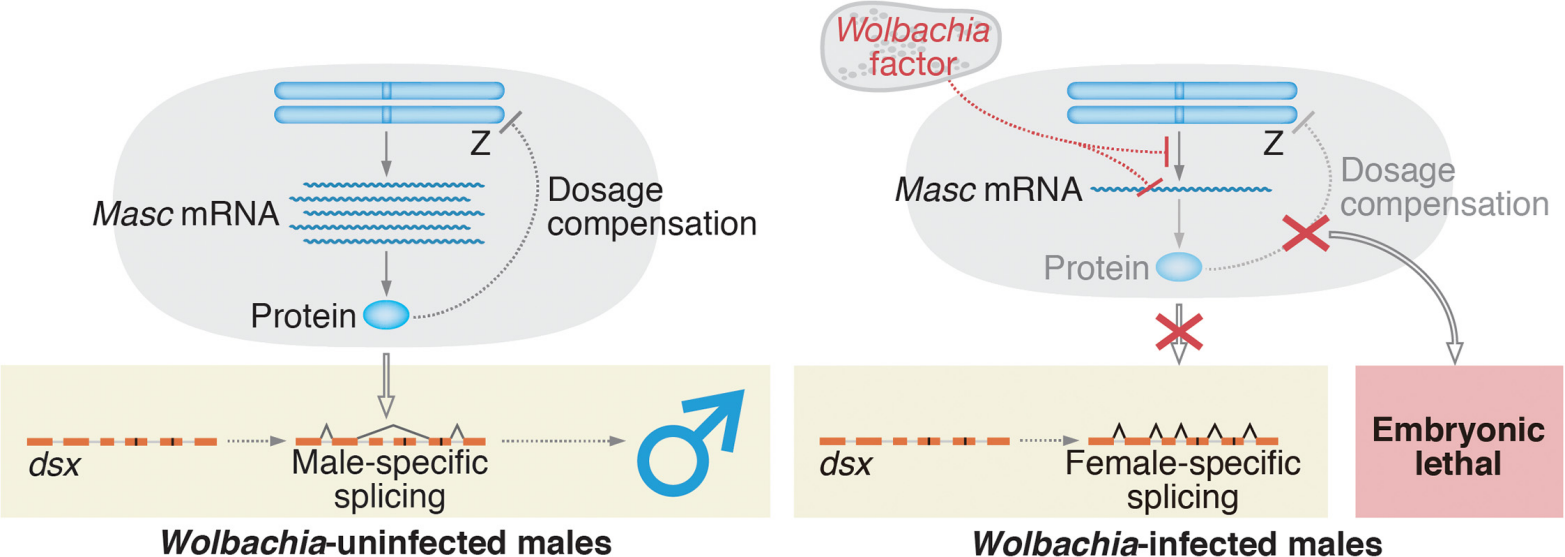
A proposed model for *Wolbachia*-induced male-killing in *Ostrinia*. In uninfected *Ostrinia* (left panel), Masc protein is expressed and utilized for dosage compensation and masculinization in male development. By contrast, in *Wolbachia*-infected *Ostrinia* (right panel), *Wolbachia* reduces *Masc* mRNA level in early embryos in an unknown manner. Down-regulation of *Masc* mRNA inhibits a masculinizing pathway, resulting in loss of the male-type variants of *dsx*. Simultaneously, dosage compensation fails to establish and male-specific embryonic lethality occurs.

In conclusion, our study answered the question of how *Wolbachia* manipulates sex ratios in moths: in *Ostrinia*, *Wolbachia* targets *Masc*, a masculinizing gene that was originally characterized in *B*. *mori*, to establish male-killing (Fig 6). In *Drosophila*, the Sex lethal protein functions as a master switch for sex determination, and also controls dosage compensation by inhibiting translation of *male-specific lethal 2* (*msl-2*) [17]. Veneti *et al*. reported that a male-killing *Spiroplasma* targets the dosage compensation complex, including *msl-2*, to kill male *D*. *melanogaster* [18]. Our current results demonstrate that a similar event occurs in *Wolbachia*-infected lepidopteran insects; *Wolbachia* infection leads to male-killing by down-regulating *Masc* (Fig 3A and 3B), which is an essential factor controlling both sex determination and dosage compensation pathways in lepidopteran insects [14]. This analogy comes from the fact that the sex determination cascade is often tightly associated with the control of dosage compensation in insects.

In *B*. *mori*, femaleness is determined by *Fem* piRNA-mediated, highly tuned post-transcriptional regulation of *Masc* mRNA [14, 19]. Our findings suggest that *Wolbachia* has captured an unknown factor through evolution and succeeded in mimicking this sex determination system to execute the male-specific death. Our future goal is to identify a *Wolbachia* factor that decreases *Masc* mRNA post-transcriptionally or that directly inhibits *Masc* transcription (Fig 6).

## Materials and Methods

### Insects

Moths were collected at Matsudo (35.8° N, 139.9° E) and Nishi-Tokyo (35.4° N, 139.3° E), Japan, in early summer, 2014. GC-MS analysis of the pheromone gland extracts of the moths used in this study showed the presence of (*E*)-12- and (*Z*)-12-tetradecenyl acetates (E12-14:OAc and Z12-14:OAc). The relative abundance of the two components was 1:1.6 (E12-14:OAc and Z12-14:OAc) in *Wolbachia*-infected and 1:2.7 in *Wolbachia*-uninfected moths, indicating that they were *O*. *furnacalis* (Lepidoptera: Crambidae). *Wolbachia*-infected strain was maintained by crossing with *Wolbachia*-free *O*. *furnacalis* male moths. *Ostrinia* larvae were reared on an artificial diet (Insecta LF, Nosan Corp.) at 23°C under a photoperiod of 16 L and 8 D. Tetracycline treatment was performed as described previously [7].

### Molecular sexing

Molecular sexing of *Ostrinia* moths and embryos was performed by qPCR using two Z-linked genes *triose phosphate isomerase* (*Tpi*) and *kettin* as described by Kern *et al*. [20]. The autosomal gene *EF-1α* was used for normalization. Primers used for qPCR are listed below:

Tpi_F: 5'-ACGGAGGATCGGTTACTGGAGC-3'
Tpi_R: 5'-CGATGTCAACGAACTCTGGCTTGA-3'
kettin_F: 5'-AGGACTCTGGACGCATGGCT-3'
kettin_R: 5'-TGCAAGGCTATCAACAGGGCA-3'
EF1a_F: 5'-TTGCCACACAGCCCACATTG-3'
EF1a_R: 5'-TTGACAATGGCGGCATCACC-3'

### RNA-seq analysis

Total RNA and genomic DNA were prepared simultaneously from *Ostrinia* embryos (25–50 embryos at each time point) using TRIzol reagent (Invitrogen) according to the manufacturer’s protocol. Libraries for RNA-seq were generated from 0, 12, 24, 36, 48 hpo embryos using the TruSeq RNA Sample Preparation kit (Illumina). The cDNAs were analyzed using the Illumina HiSeq 2500 platform with 100-bp paired-end reads according to the manufacturer's protocol [21]. *De novo* assembly of RNA-seq data from 10 data sets was performed as described previously [14]. *Ostrinia Masc* was identified from assembled contigs by BLAST using the *B*. *mori* Masc amino acid sequence as a query.

Because extensive synteny conservation is observed among several lepidopteran insects including *Ostrinia* [22–24], we identified putative corresponding chromosomes for *Ostrinia* RNA-seq derived contigs by BLAST using 13,789 *B*. *mori* gene models (putative protein-coding genes whose chromosomal locations are identified). Transcript abundance in each contig was quantified as described previously [14–15]. RNA-seq data of *B*. *mori Masc* RNAi experiments (*GFP* and *Masc* RNAi embryos of each sex, 72 h post-injection, 4 data sets) [14] were used as a control data set.

### RT-PCR

Total RNA and genomic DNA were prepared from *Ostrinia* embryos (25–50 embryos at each time point) using TRIzol reagent (Invitrogen) according to the manufacturer’s protocol. Total RNA was subjected to reverse transcription using avian myeloblastosis virus (AMV) reverse transcriptase with an oligo-dT primer (TaKaRa). PCR was carried out with KOD FX-neo DNA polymerase (TOYOBO). Sex-specific splicing of *Ostrinia dsx* by RT-PCR and *Wolbachia* detection by *wsp* PCR were performed with primers reported previously [6]. RT-qPCR analyses were performed using a KAPA^TM^ SYBR FAST qPCR kit (Kapa Biosystems) and specific primers. The expression levels of *rps3* were used to normalize transcript levels. Primers used for RT-qPCR are listed below:

OstriniaMasc_F: 5'-TTTGCCGCATTCATTCGCAG-3'
OstriniaMasc_R: 5'-TGGTTTTGGTGCAAGCAATTCG-3'
OstriniaRPS3_F: 5'-TGGCCACCAGAACTCAAAGC-3'
OstriniaRPS3_R: 5'-GAAACGCTTCTGGACTACGGA-3'

### Transfection of *Ostrinia Masc* cDNA in BmN4 cells

*Ostrinia Masc* cDNA was cloned into the pIZ/V5-His vector (Invitrogen). Transfection experiments were performed as described previously [14]. In brief, BmN4 cells were transfected with plasmid DNAs using X-tremeGENE HP (Roche). Cells were collected at 72 h after transfection, and RT-PCR for *Bmdsx* and RT-qPCR for *BmIMP* were performed. *B*. *mori Masc* was used as a positive control for this experiment. *BmIMP* mRNA level was normalized to that of *rp49*. Primers used for RT-qPCR are listed below:

Bmdsx_F: 5'-AACCATGCCACCACTGATACCAAC-3'
Bmdsx_R: 5'-GCACAACGAATACTGCTGCAATCG-3'
rp49_F: 5'-CCCAACATTGGTTACGGTTC-3'
rp49_R: 5'-GCTCTTTCCACGATCAGCTT-3'
BmIMP_F: 5'-ATGCGGGAAGAAGGTTTTATG-3'
BmIMP_R: 5'-TCATCCCGCCTCAGACGATTG-3'

### cRNA injection

The DNA template for cRNA synthesis was amplified by PCR using the pIZ/V5-His vector containing *Ostrinia Masc* (described above) or *GFP* (control) cDNA. Primers used for PCR are listed below:

pIZ-F-T7: 5'-TAATACGACTCACTATAGGGAGACAGTTGAACAGCATCTGTTC-3'
pIZ-R: 5'-GACAATACAAACTAAGATTTAGTCAG-3'

Capped, poly(A)-tailed cRNA was synthesized using mMESSAGE mMACHINE T7 Ultra Kit (Ambion). cRNA injection was performed as described previously [14] with some modifications. We injected 1–2 nl of *Masc* or *GFP* cRNA solution (1 μg/μl in 100 mM potassium acetate, 2 mM magnesium acetate, 30 mM HEPES-KOH; pH7.4) into *Wolbachia*-infected *Ostrinia* embryos within 4 h after oviposition. The hatched larvae were collected and molecularly sexed by qPCR.

### Phylogenetic analysis

Phylogenetic analysis of *Ostrinia* Masc protein was performed as described previously [14]. The amino acid sequences of proteins in the NCBI database and those deduced form the RNA-seq data obtained in this study with significant homology (E-value of < 1 × 10^−9^) to residues 51–122 of Masc were identified using the BLAST program. A neighbor-joining tree was constructed using 43 sequences [including 3 *Ostrinia* sequences (*Ostrinia Masc*, c66984_g1_i4, and c66984_g1_i3)] and the reliability of the tree was tested by bootstrap analysis with 1000 replications.

### Sequence deposition

The nucleotide sequence of *Ostrinia Masc* has been submitted to the DDBJ/EMBL/GenBank data bank under the accession number LC028928. Deep sequencing data obtained in this study are available under the accession number DRA003038 (DDBJ).

## Supporting Information

S1 Fig Characterization of a male-killing *Wolbachia* found in *O*. *furnacalis*. (A) Establishment of the molecular sexing method for *O*. *furnacalis*. Molecular sexing of *Wolbachia*-uninfected *O*. *furnacalis* moths was performed by qPCR using two Z-linked genes *Tpi* and *kettin*. The autosomal gene *EF-1α* was used for normalization. Moth sexes were judged by external morphology. The number indicates the sample size of each group. (B) Molecular sexing of *Wolbachia*-infected *O*. *furnacalis* moths. The number indicates the sample size of each group. (C) Molecular sexing of *Wolbachia*-infected embryos (just prior to hatching, left panel) or hatched larvae (right panel). The number indicates the sample size of each group. (D) Brood sex ratios in a *Wolbachia*-eliminated matriline by tetracycline treatment. The female:male ratio of two independent mating is shown. (E) Splicing patterns of *Ostrinia dsx* in *Wolbachia*-free embryos at 60 hpo. The female-type splicing variants were clearly detected from the same RNA pool used in Fig 1C.
(EPS)

S2 Fig Identification of *Ostrinia* Masc protein. Alignment of *B*. *mori* and *Ostrinia* Masc proteins was performed by the Clustal W program. The locations of putative zinc finger domains are indicated by red lines.
(EPS)

S3 Fig MA plots of RNA-seq data of *Wolbachia*-infected and uninfected *Ostrinia* during embryogenesis. The *Ostrinia* contigs that were assigned to *B*. *mori* gene models were used for analysis. Z-linked genes are indicated by red dots.
(EPS)
